# Supplementary material for: Properties of Putative APSES Transcription Factor AfpA in Aspergillus fumigatus
Source: J Fungi (Basel). 2025 Sep 16;11(9):678. doi: 10.3390/jof11090678 (PMC12470788; doi:10.3390/jof11090678)
Supplement: Supplementary file 1 [file jof-11-00678-s001.zip › Fig. S1.pptx]

## Slide 1
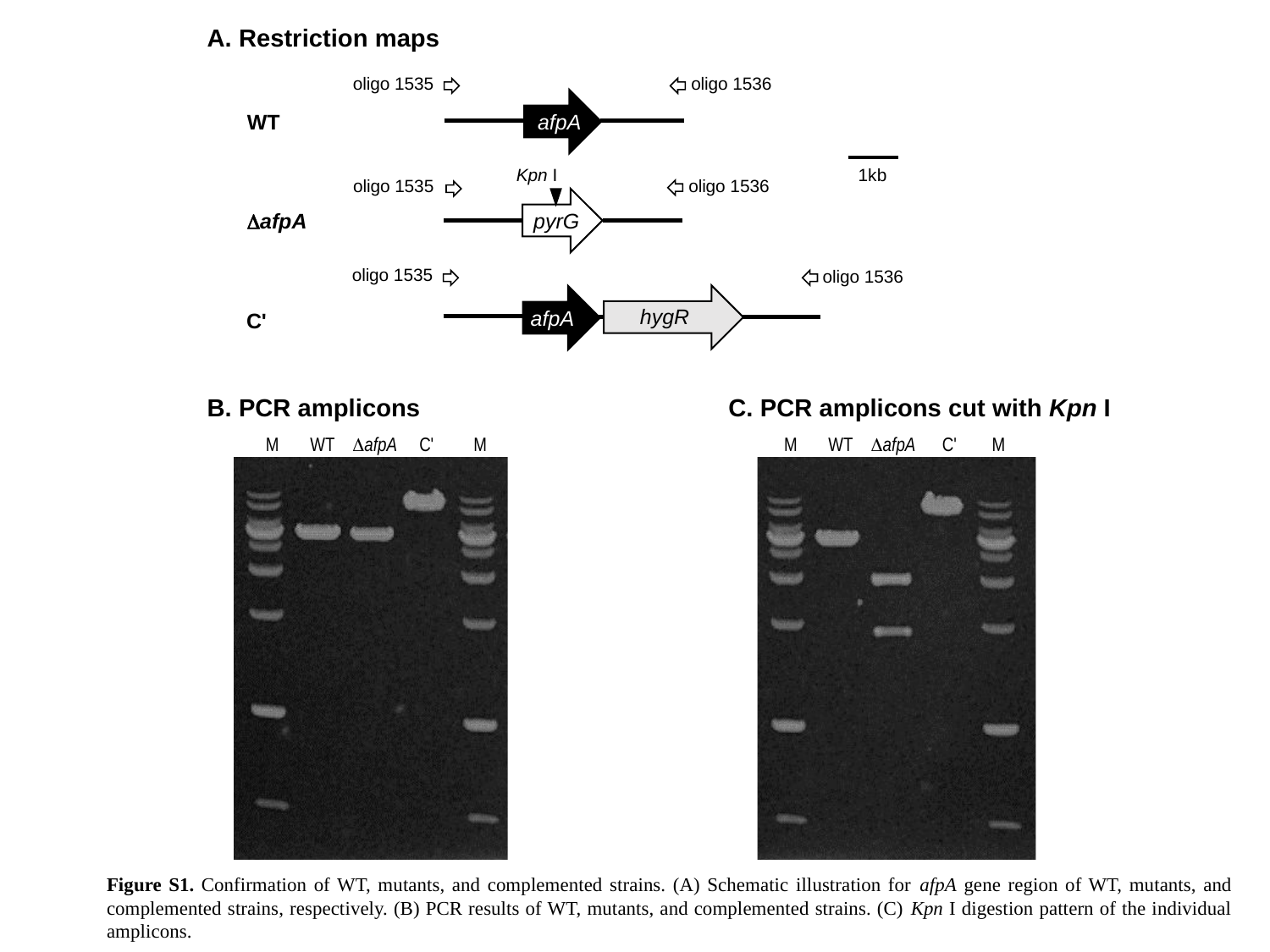

A. Restriction maps
oligo 1535
oligo 1536
afpA
WT
1kb
Kpn I
oligo 1535
oligo 1536
pyrG
DafpA
oligo 1535
oligo 1536
hygR
afpA
C'
B. PCR amplicons
C. PCR amplicons cut with Kpn I
M WT DafpA C' M
M WT DafpA C' M
Figure S1. Confirmation of WT, mutants, and complemented strains. (A) Schematic illustration for afpA gene region of WT, mutants, and complemented strains, respectively. (B) PCR results of WT, mutants, and complemented strains. (C) Kpn I digestion pattern of the individual amplicons.
