# Supplementary material for: Properties of Putative APSES Transcription Factor AfpA in Aspergillus fumigatus
Source: J Fungi (Basel). 2025 Sep 16;11(9):678. doi: 10.3390/jof11090678 (PMC12470788; doi:10.3390/jof11090678)
Supplement: Supplementary file 1 [file jof-11-00678-s001.zip › Fig. S2.pptx]

## Slide 1
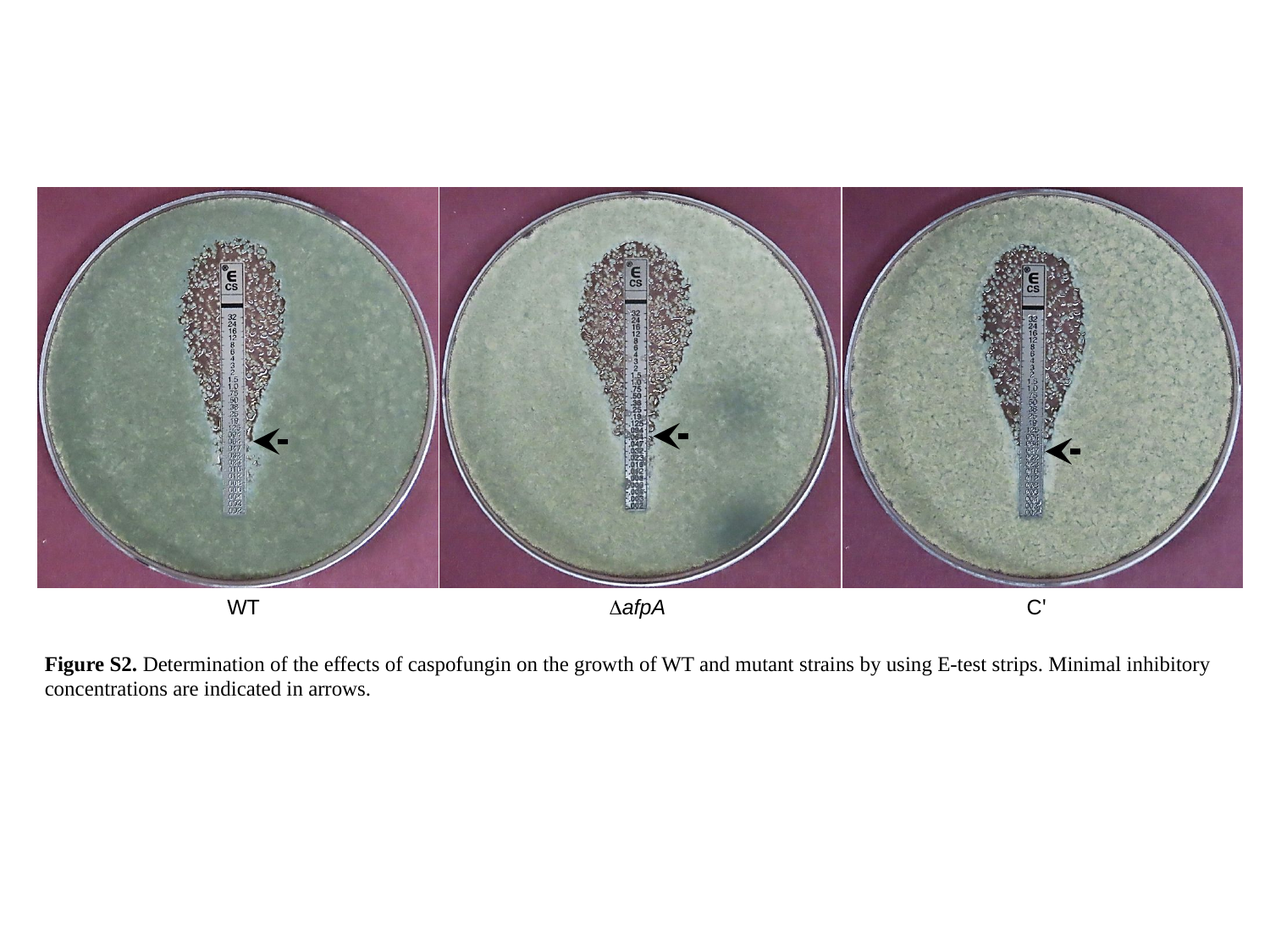

WT DafpA C'
Figure S2. Determination of the effects of caspofungin on the growth of WT and mutant strains by using E-test strips. Minimal inhibitory concentrations are indicated in arrows.
