# Supplementary material for: Properties of Putative APSES Transcription Factor AfpA in Aspergillus fumigatus
Source: J Fungi (Basel). 2025 Sep 16;11(9):678. doi: 10.3390/jof11090678 (PMC12470788; doi:10.3390/jof11090678)
Supplement: Supplementary file 1 [file jof-11-00678-s001.zip › Table S1.pdf]

Table S1. Oligonucleotides used in this study.

Name	Sequence (5'→3')[a]	Purpose
oligo 256	TTCCAAGCAGAGCTTGTCAC	5' *brlA* for RT-qPCR
oligo 257	CCAGGTTCTTTGCACTTGAA	3' *brlA* for RT-qPCR
oligo 277	ATTCCAGAGGAGAAGCAGGA	5' *catA* for RT-qPCR
oligo 278	GAGCTCTCCAATCACACGAA	3' *catA* for RT-qPCR
oligo 303	GCTACCACTCTGCATCCTCA	5' *abaA* for RT-qPCR
oligo 304	TACGAGCTCCAGCATGATTC	3' *abaA* for RT-qPCR
oligo 305	ACGGCAGGAAGTTGTCTTCT	5' *wetA* for RT-qPCR
oligo 306	CTGTCAGCGACTTGTTGGAT	3' *wetA* for RT-qPCR
oligo 346	CCATGTGTGTCGAGTCCTTC	5' *ef1α* for RT-qPCR normalization
oligo 347	GAACGTACAGCAACAGTCTGG	3' *ef1α* for RT-qPCR normalization
oligo 430	TTCCAAATGTGGCAAGTGAT	5' *acyA* for RT-qPCR
oligo 431	GCAAACGTGGAATCAATACG	3' *acyA* for RT-qPCR
oligo 434	CCACCACCTACAACAACAGC	5' *pkaC1* for RT-qPCR
oligo 435	TGTGAAGACGCATGATGAGA	3' *pkaC1* for RT-qPCR
oligo 1260	TAGCGGAAATGCCAACGGAGGATA	5' *chsB* for RT-qPCR
oligo 1261	GAGAAGCAGGGCGCGATGAATACT	3' *chsB* for RT-qPCR
oligo 1262	ATCGCTGCATTCCTGGGTCTGTCG	5' *chsE* for RT-qPCR

oligo 1263	CGCGCAAGCTCATCGGCATTTT	3' *chsE* for RT-qPCR
oligo 1531	GGGCATAGGCAGAGATACCA	5' flanking region of *afpA*
oligo 1532	*CTGATCTACCCCTTGGAACGCAGCA*TGAAGGATGTGACTGCGCCT	3' *afpA* with *AnpyrG* tail
oligo 1533	*TTTGTAGGCTTTGGGCTGTTCACAA*AATCTAGTCCCGGCTCTTCC	5' *afpA* with *AnpyrG* tail
oligo 1534	CCCGACCTTAGTCGCATATG	3' flanking region of *afpA*
oligo 1535	CAAGATCATGAGAAGGGCGG	5' nested of *afpA*
oligo 1536	CAGAATCTTCGCCACGCTC	3' nested of *afpA*
oligo 1667	GTGCGCGCTATCCAAACAAT	5' *gfaA* for RT-qPCR
oligo 1668	ACGACTGACTCGGGTTGTTC	3' *gfaA* for RT-qPCR
oligo 1761	*TAGTTCTGTTACCGAGCCGG*GGA AGA GCC GGG ACT AGA TT	5' *afpA* with *hygB* tail for C'
oligo 1762	*GCTCTGAACGATATGCTCCAAC*GCC AGG AGC AGA GTA TGA TT	3' *afpA* with *hygB* tail for C'

[a] Tail sequence is in italic.
