# Supplementary material for: Properties of Putative APSES Transcription Factor AfpA in Aspergillus fumigatus
Source: J Fungi (Basel). 2025 Sep 16;11(9):678. doi: 10.3390/jof11090678 (PMC12470788; doi:10.3390/jof11090678)
Supplement: Supplementary file 1 [file jof-11-00678-s001.zip › Table S2.pdf]

Table S2. Representative up-regulated genes in Δ*afpA* relative to WT strain ($p < 0.05$).

**Gene symbol**	**Fold change**	***p* value**	**Product**
AFUA_4G08840	24.0888685	0.020843686	RING finger protein, putative
AFUA_5G14030	18.07989086	7.71478E-05	endo-1,3(4)-beta-glucanase, putative
AFUA_1G13220	15.57828522	0.044508787	cytochrome P450 pisatin demethylase, putative
AFUA_6G02490	14.61387066	2.53612E-05	aspartate transaminase, putative
AFUA_5G04155	14.34011347	0.011703249	conserved hypothetical protein
AFUA_1G16360	13.42158694	0.01619885	hypothetical protein
AFUA_4G14740	13.28343691	0.002184396	serine/threonine protein kinase (Ark1), putative
AFUA_5G02280	13.23618677	0.022576705	endo-1,3(4)-beta-glucanase, putative
AFUA_4G11750	13.21771899	0.036184939	conserved hypothetical protein
AFUA_2G09590	13.21568331	0.015349813	UDP-N-acetylglucosamine 1-carboxyvinyltransferase family protein
AFUA_8G02040	12.76548975	0.047839099	glycosyl transferase, putative
AFUA_6G09690	12.555342	0.001353635	glutathione S-transferase GliG
AFUA_8G02760	12.29813561	0.043833587	mitochondrial ornithine carrier protein AmcA/Ort1, putative
AFUA_6G08390	12.007962	0.022404523	conserved hypothetical protein
AFUA_1G05260	11.42801247	0.048833231	DNA-directed DNA polymerase theta, putative
AFUA_7G06200	11.14465395	0.034486235	ankyrin repeat protein
AFUA_2G11330	11.04774431	0.029223968	V-type ATPase F subunit, putative
AFUA_4G11970	10.57008395	0.001257666	metallothionein-I gene transcription activator
