# Supplementary material for: Properties of Putative APSES Transcription Factor AfpA in Aspergillus fumigatus
Source: J Fungi (Basel). 2025 Sep 16;11(9):678. doi: 10.3390/jof11090678 (PMC12470788; doi:10.3390/jof11090678)
Supplement: Supplementary file 1 [file jof-11-00678-s001.zip › Table S3.pdf]

Table S3. Representative down-regulated genes in Δ*afpA* relative to WT strain ($p < 0.05$).

**Gene symbol**	**Fold change**	***p* value**	**Product**
AFUA_3G03330	0.087157471	0.002650031	mitochondrial enoyl reductase, putative
AFUA_3G13780	0.092686068	0.001857124	conserved hypothetical protein
AFUA_5G06830	0.096218967	0.000198883	dsRNA-specific ribonuclease III, putative
AFUA_4G04020	0.096753765	0.039336619	FKBP-type peptidyl-prolyl isomerase, putative
AFUA_6G12020	0.127896021	0.01055207	C2H2 finger domain protein, putative
AFUA_6G03130	0.140987653	0.00236293	hypothetical protein
AFUA_7G00160	0.143654603	0.011206409	polyketide synthase, putative
AFUA_4G06570	0.146445761	6.21581E-05	Ras guanine-nucleotide exchange protein, putative
AFUA_3G01470	0.148037108	4.75706E-05	conserved hypothetical protein
AFUA_2G00300	0.152426299	0.000251561	hypothetical protein
AFUA_7G00210	0.160887199	0.01173369	C6 transcription factor, putative
AFUA_6G09790	0.162946889	0.001784153	LysM domain protein, putative
AFUA_5G01260	0.163559871	0.011987621	ankyrin repeat protein
AFUA_7G00290	0.164457159	0.034026015	cytochrome P450 monooxygenase, putative
AFUA_1G17650	0.165530333	0.00567114	short chain dehydrogenase, putative
AFUA_8G02630	0.167471992	0.014276867	extracellular exo-polygalacturonase, putative
AFUA_8G06070	0.168868937	0.03409349	hypothetical protein
AFUA_7G04340	0.190996931	0.042552954	C6 transcription factor, putative
